# Do different dental conditions influence the static plantar pressure and stabilometry in young adults?

**DOI:** 10.1371/journal.pone.0228816

**Published:** 2020-02-11

**Authors:** Elena Amaricai, Roxana Ramona Onofrei, Oana Suciu, Corina Marcauteanu, Eniko Tunde Stoica, Meda Lavinia Negruțiu, Vlad Laurentiu David, Cosmin Sinescu

**Affiliations:** 1 Department of Rehabilitation, Physical Medicine and Rheumatology, “Victor Babes” University of Medicine and Pharmacy, Timisoara, Romania; 2 Department of Occlusiology, Faculty of Dentistry, “Victor Babes” University of Medicine and Pharmacy, Timisoara, Romania; 3 Dental Materials and Dental Prosthesis Department, Faculty of Dentistry, “Victor Babes” University of Medicine and Pharmacy, Timisoara, Romania; 4 Department of Pediatric Surgery and Orthopedics, “Victor Babes” University of Medicine and Pharmacy, Timisoara, Romania; West Virginia University, UNITED STATES

## Abstract

**Background:**

Posture is influenced by many factors and dental occlusion seems to have its role on postural stabilization. Our rationale to perform the study was to find out if there are differences of static plantar pressure and stabilometric parameters depending on different dental conditions.

**Methods:**

The observational study consisted in plantar pressure assessment and stabilometric analysis of 95 right-handed healthy volunteer subjects (mean age 22.94 ± 2.52 years) by using the PoData system. Each subject followed four measurements with open eyes: mandibular postural position, maximum intercuspation, biting on cotton rolls and maximum mouth opening. Plantar pressure was recorded on 1^st^ and 5^th^ metatarsal heads and heel, and was expressed as percentage of weight distribution on each foot. The recorded centre of pressure (CoP) parameters were: CoP path length, 90%confidence ellipse area and maximum CoP speed. Statistical analysis used repeated-measures ANOVA with Bonferroni posthoc analysis and Friedman test.

**Results:**

Loading on the left 5^th^ metatarsal head was significantly higher in maximum mouth opening condition when compared to maximum intercuspation and to biting on cotton rolls. The left heel loading was significantly lower in the maximum mouth opening in comparison to maximum intercuspation. The CoP path length and maximum CoP speed were significantly higher in maximum mouth opening compared to the other three conditions. Confidence ellipse area had significantly lower values in maximum intercuspation and in the biting on cotton rolls conditions compared to the mandibular postural position, and in maximum intercuspation compared to maximum mouth opening.

**Conclusion:**

In young adults with an optimum functional occlusion the static plantar pressure is influenced by the maximum mouth opening. An improved postural stability was recorded in maximum intercuspation (a condition used during swallowing) in comparison to mandibular postural position (a condition that allows relaxation of the masticatory muscles after functional moments).

## Introduction

The posture is influenced by many factors, while dental occlusion seems to have its role on postural stabilization [1]. Many reviews have analysed the influence of dental occlusion on body balance, focusing on the neuroanatomical interconnections between the trigeminal and vestibular nuclei, and on the muscular interconnections implying influences between the masticatory and the postural muscles. Moon and Lee showed that dental occlusion influences body-stabilizing activities such as body equilibrium and gravity fluctuation [2]. The review of Cuccia and Caradonna also suggested that there are correlations between posture and the stomatognathic system [3]. Moreover, changes of different jaws relations also have effect on postural stability [4]. A more recent study (2013) demonstrated a weak correlation between mandibular position and body posture in healthy subjects [5].

In contrast with the previous findings, the review of Manfredini D. et al. showed that there is no evidence for the existence of a predictable relationship between occlusal and postural features [6]. The review of Perinetti G. from 2013 analysed 11 studies regarding the effects of mandibular position on body sway. The results were different, but the general conclusion was that the mandibular position does not appear to be correlated to body sway at a clinically significant level [7]. Another overview stated that it is not recommended to perform occlusal treatment in order to treat or prevent postural imbalances or alteration of spine curvatures [8]. The study of Perinetti G. from 2006 showed no detectable correlation at the posturography level between dental occlusion and body posture. However, the existence of a correlation between these structures cannot be denied [9].

In spite of these contrasting results, a recent review of Julia-Sanchez S. et al. (2019) stated that more studies are needed to elucidate the mechanisms by which dental occlusion may influence balance control. The main conclusion of this review was that dental occlusion contributes to balance control when there are external perturbations such as unstable support surface or during performing unipedal or bipedal tasks [10].

The relationship between dental occlusion and posture was also studied in subjects with temporo-mandibular disorders, as well as in physically well-trained or sporty subjects. The study of Nota A. et al. demonstrated a significant difference in body postural stability between subjects with myogenous temporo-mandibular disorders and healthy controls, with increased sway area and sway velocity postural parameters in the first group [11]. Dental occlusion can influence posture in air force and civilian pilots [12], while repercussion of dental occlusion upon posture stabilization is suggested in the high level shooters [13].

Our rationale to perform the current study was to find out if there are differences of static plantar pressure and stabilometric parameters depending on different dental conditions. To our knowledge there are no studies that compared the distribution of the plantar pressure on the right and left foot, as well as the three weight distribution sites of each foot (1^st^ and 5^th^ metatarsal heads, and calcaneus) in relation with dental occlusion.

The objective of our study was to analyse the distribution of static plantar pressure and stabilometric parameters in relation to different dental conditions in healthy young adults.

## Material and methods

### Subjects

One hundred twenty-eight healthy volunteer subjects were asked to participate in the study. They were recruited among the graduates of our university, their friends, relatives or acquaintances. The sample size was calculated using G*Power software version 3.1.9.2. For α = 0.05, β = 0.2, a medium effect size (f = 0.25), with four measurements, a sample size of at least 94 subjects would be required [14].

The subjects included in this study underwent a detailed anamnesis and a thorough extraoral and intraoral examination. The inclusion criteria were: age over 18 years; presence of at least 28 teeth, with no large occlusal restorations (the composite and/or amalgam fillings satisfied the criteria of an optimum functional occlusion according to Okeson [15]); class I interincisal relationships (with an overbite of 2–4 mm and an overjet of 1–2 mm); correct Angle’s key on both sides. The exclusion criteria were: foot disorders; spinal deviations; myogenous or arthrogenous temporo-mandibular disorders; trauma or surgery that can influence posture; history of neurological diseases, vestibular or visual disturbances or any other pathology that would influence posture; any type of crossbite, open bite or deep bite; extensive occlusal restorations through either large fillings or fixed partial dentures. The TMD patients were excluded from this study because of the significant difference in body posture between subjects with TMD and healthy controls [11].

Participation in the study was voluntary. Written informed consent was obtained from all the subjects. The study has been carried out in accordance with the Declaration of Helsinki and was approved by Institutional Ethics Committee (no 23/2019).

### Assessments

PoData system (Chinesport, Udine, Italy) was used for plantar pressure assessment and stabilometric analysis. The PoData is a capacitive pressure distribution system with an integrated podoscope and six load cells. The data are recorded at a sampling frequency of 100 Hz and analysed by the provided GPS5 software. We followed the manufacturer’s instructions, performing an initial no-load calibration of the platform. The six virtual sensors were set after the subjects step up on the platform, corresponding to the 1^st^ and 5^th^ metatarsal heads and heel, in both right and left foot [16]. The system provides information about weight distribution, barycentre and stabilometry [17].

The subjects were asked to stand on the platform, barefoot, in upright posture, lower limbs extended and arms positioned naturally along their sides. The feet were positioned at an angle of 30° to each other and 5 cm between the heels [18].

They were instructed to look ahead, fixing a target point on the wall, not to talk or move (S1 Fig). Measurements of plantar pressure and stabilometry were performed for each subject under the following conditions, with open eyes: mandibular postural position (the position of the mandible when an individual is resting comfortably in an upright position, with the condyles in a neutral, unstrained position in the glenoid fossae and the associated muscles are in a state of minimal contractual activity [19]), maximum intercuspation (optimum occlusal stabilization), biting on three 8-mm cotton rolls placed between the opposing teeth at the level of the 1^st^ molars and central incisors, and maximum mouth opening. The mandibular postural position is characterized by a balance between the tonus of the mandibular elevator muscles and the tonus of the mandibular depressor muscles; it is maintained by the intervention of the myotactic reflex and is adopted by the mandible after swallowing saliva [15]. The postural position of the mandible was confirmed by the following issues: the patient`s lips were touching lightly and effortlessly; the postural vertical dimension (measured between *subnasale* and *gnathion* with the Willis bite gauge) was equal to the distance between the corner of the mouth and the external angle of the eye; there was a space of 2–4 mm between the opposing dental arches (freeway space). Each recording had duration of 20 seconds. The evaluations were performed by the same experienced investigator. The testing was considered invalid and repeated if at least one of the following errors were observed: the subject moved or lifted an arm or both, lifting the forefoot or the heel, falling out of position, moving the head or talking.

Plantar pressure was recorded on three anatomical regions: 1^st^ and 5^th^ metatarsal heads and heel, in both right and left foot [20]. Percentage of body weight distribution was calculated for each area. An ideal load of an ideal subject has the following distribution: 1/6 (16.7%) of total weight on 5^th^ metatarsal head, 2/6 (33.33%) of total weight on 1^st^ metatarsal head, and 3/6 (50%) of total weight on calcaneus, for the right and the left foot, respectively [17].

The measured body centre of pressure (CoP) was compared with the theoretical one [21,22]. Body centre of pressure deviation from theoretical reference was measured on anterior-posterior (CoP_Y_) and latero-lateral (CoP_X_) axes [17,21,23]. Average distances from ideal barycentre were provided by the software for both axes. A positive value represents an anterior deviation on the anterior-posterior axis, and a right deviation on the latero-lateral axis. Based on these deviations, the absolute mean CoP displacement from the ideal position was calculated [9]. Other CoP parameters analysed in this study were the CoP path length, the 90% confidence ellipse area and the maximum CoP speed, as recommended by Nagymate and Kiss [24]. The CoP path length is the length in millimetres of the subject’s centre of gravity shift during the test. Confidence ellipse area is the area in mm^2^ of the ellipse that includes all the centre of gravity points measured and transferred on a system of Cartesian axes with a confidence level of 90%. Maximum CoP speed is the average centre of gravity shifting maximum speed in millimetres per second.

### Statistical analysis

Statistical analysis was performed using MedCalc version 8.11 (MedCalc Software bvba, Ostend, Belgium). Data were tested for normality with the Shapiro-Wilk test, and presented as mean and standard deviation for normal distributed data, and as median and interquartile range for non-normally distributed data. Differences in weight distribution and stabilometric parameters among the four conditions were assessed using the repeated-measures ANOVA with a Bonferroni posthoc analysis and a Friedman test, respectively. Statistical significance was set p<0.05 for all tests.

## Results

Ninety-five subjects (66 females and 29 males, mean age 22.94 ± 2.52 years) met the inclusion criteria and their data were analysed. The demographic characteristics are presented in Table 1. All the subjects were right-handed.

**Table 1:** Subjects’ demographic characteristics.

Variables	
**Age**, years (mean ± SD)	22.94 ± 2.52
**Gender**	
** Male**, n (%)	29 (30.5)
** Female**, n (%)	66 (69.5)
**Weight**, kg (mean ± SD)	64.17 ± 12.51
**Height**, cm (mean ± SD)	168.02 ± 8.23
**BMI** (mean ± SD)	22.61 ± 3.71

n: number of subjects; SD: standard deviation; BMI: body mass index.

The weight distributions in all the testing conditions are presented in Table 2. Significant main effects of the conditions were found for the left 5^th^ metatarsal head (_F3,282_ = 4.26, p = 0.006) and left heel weight distribution (F_3,282_ = 3.72, p = 0.011). Posthoc analysis showed that loading on the left 5^th^ metatarsal head was significantly higher in the maximum mouth opening condition when compared to maximum intercuspation (p = 0.003) and to biting on cotton rolls (p = 0.04). The left heel loading was significantly lower in the maximum mouth opening condition in comparison to maximum intercuspation (p = 0.003).

**Table 2:** Static plantar pressure load distribution.

Variables	Mandibular postural position	Maximum intercuspation	Bitingon cotton rolls	Maximum mouth opening
**Right foot** (%)	49.85 ± 3.87	49.47 ± 4.61	49.43 ±4.83	49.71 ± 4.69
**Right MT1** (%)	19.92 ± 8.08	19.69 ± 8.61	19.62 ± 8.93	19.63 ± 8.87
**Right MT5** (%)	37 ± 8.29	36.72 ± 8.63	36.92 ± 8.11	37.24 ± 9.61
**Right heel** (%)	43.19 ± 10.85	43.68 ± 10.83	43.25 ± 10.46	43.23 ± 11.84
**Left foot** (%)	50.15 ± 3.87	50.49 ± 4.57	50.57 ± 4.83	50.29 ± 4.69
**Left MT1** (%)	22.08 ± 6.62	21.51 ± 7.06	21.88 ± 6.68	22.36 ± 7.38
**Left MT5** (%)	31.62 ± 9.13	30.81 ± 9.05	30.97 ± 9.47	32.05 ± 9.41
**Left heel** (%)	46.42 ± 11.08	47.63 ± 11.33	47.07 ± 11.85	45.73 ± 11.69

Data are presents as mean ± standard deviation; MT1: 1^st^ metatarsal head; MT5: 5^th^ metatarsal head.

**Fig 1:**
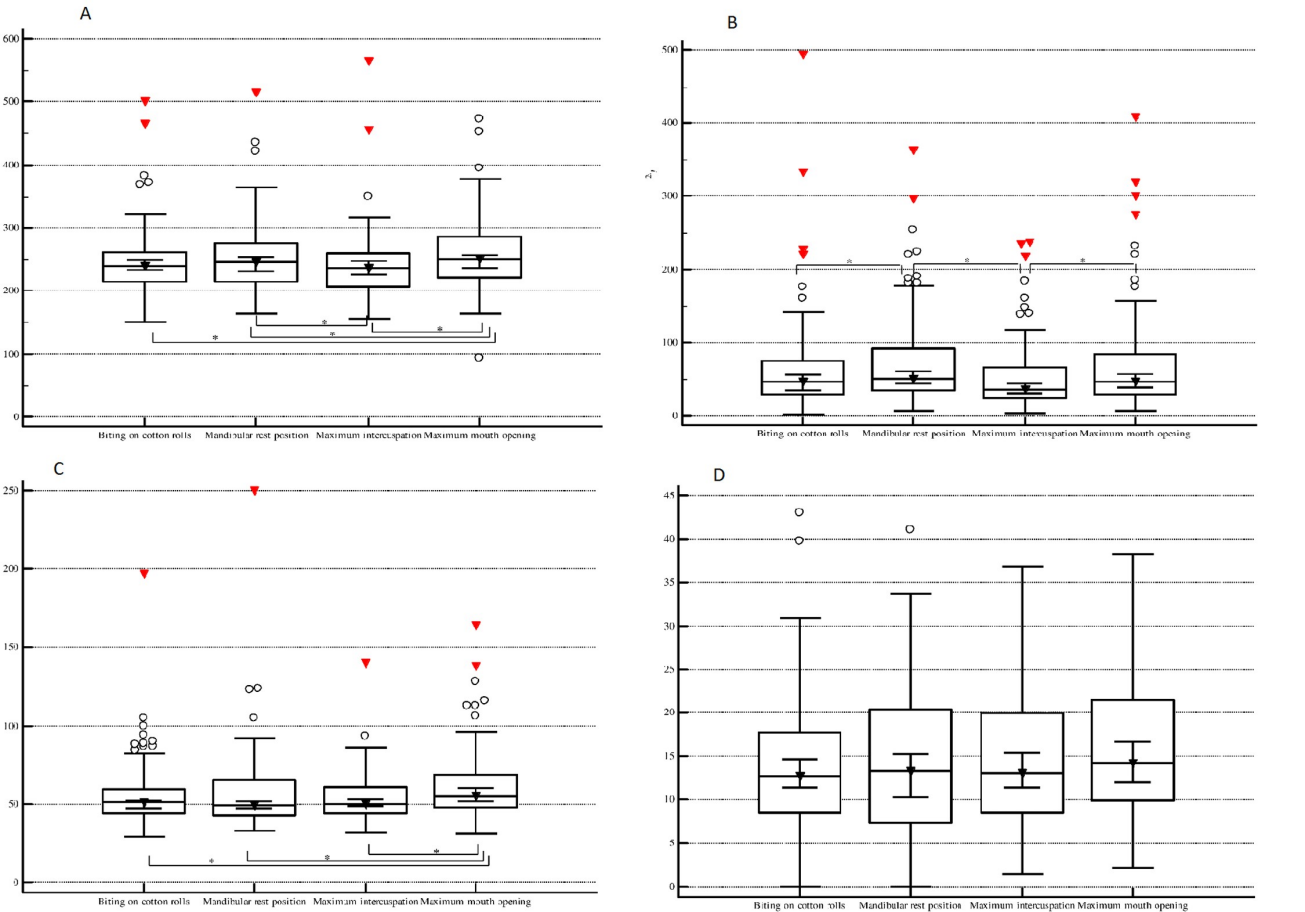
Stabilometric data comparison. Box plots (median, 95% confidence interval, interquartile range) describe the COP path length (A), 90% confidence area (B), maximum COP speed (C) and COP displacement (D) according to the condition. (*—p<0.05).

The stabilometric data are presented in Table 3 and Fig 1A–1D. Significant main effects of the conditions were observed for CoP path length (F_3,282_ = 7.07, p = 0.0001), 90% confidence ellipse area (F_3,282_ = 5.36, p = 0.001) and maximum CoP speed (F_3,282_ = 4.55, p = 0.003). The CoP path length was significantly higher in maximum mouth opening compared to all the other conditions (p<0.05). This parameter was significantly lower in maximum intercuspation compared to the mandibular postural position (p<0.05). When recording the 90% confidence ellipse area we noticed that it had significantly lower values in maximum intercuspation and in the biting on cotton rolls conditions compared to the mandibular postural position (p<0.05), and in maximum intercuspation compared to maximum mouth opening (p<0.05). The maximum CoP speed was significantly greater in maximum mouth opening compared to the other three conditions (p<0.05). The CoP displacement did not differ significantly across conditions.

**Table 3:** Stabilometric data.

	Mandibular postural position	Maximum intercuspation	Biting on cotton rolls	Maximum mouth opening	p
**CoP**_**X**_ *	0.97 ± 7.91	1.29 ± 9.38	0.75 ± 9.83	1.38 ± 9.88	NS
**CoP**_**Y**_ *	3.27 ± 14.17	1.42 ± 13.81	2.27 ± 12.81	2.94 ± 13.99	NS
**CoP displacement** (mm)**	13.34 [7.36–20.45]	13 [8.56–19.98]	12.73 [8.5–17.66]	14.21 [9.89–21.39]	NS
**CoP path length** (mm)**	245 [215–275]	237 [206–260.25]	239 [215.25–261.25]	250 [221–286]	0.0001
**90% confidence ellipse area** (mm^2^)**	51 [34.5–92.25]	36 [25–66]	46 [28–74.5]	47 [29–84.75]	0.001
**Maximum CoP speed** (mm/s)**	49 [43–65.5]	50 [44–61]	51 [44–59.75]	55 [48–68.75]	0.003

* Data are presented as mean ± standard deviation;

** Data are presented as median [interquartile range]; COP: centre of pressure; NS: not significant.

## Discussions

The current study aimed to investigate the static plantar pressure and stabilometry in relation to different dental conditions. We assessed young adults with an optimum functional occlusion according to Okeson [15].

The novelty of our study is represented by the fact that it assessed the static plantar pressure distributed on the 1^st^ and 5^th^ metatarsal heads and calcaneus, of the right and the left foot, in different dental conditions, namely mandibular postural position, maximum intercuspation, biting on cotton rolls and maximum mouth opening. All the examinations were performed with eyes open. We noticed that the left heel loading was reduced with an average of 1.9% in maximum mouth opening condition when compared to maximum intercuspation. In contrast, the left 5^th^ metatarsal loading increased with an average of 1.08% and 1.24%, respectively, in maximum mouth opening condition when compared to biting on cotton rolls and maximum intercuspation, respectively. Our study also compared the stabilometric parameters among the above mentioned dental conditions. Previous studies assessed the reliability of stabilometric analysis using a force platform and showed good intra- and intersession reliability [25,26]. Path length is considered to be a valid outcome measurement in numerous balance conditions; the smaller the path length, the better is the postural stability [27]. When referring to confidence area, a smaller surface means a better performance [28]. Velocity reflects the efficiency of the postural control system (the smaller the velocity, the better the postural control) [29]; it is considered the measurement with the greatest reliability among trials [30].

The study of Perinetti (2006) including 26 healthy subjects (mean age 26.8 ± 5.3 years) showed that there were no differences of the posturographic parameters (absolute mean displacement of the centre of pressure from the theoretical point, projected sway area and sway length) between mandibular postural position and dental intercuspidation [9]. Sway area refers to the swept area that connects the mean point of the trajectory to all subsequent points in the trajectory, normalised to the duration of acquisition; it is expressed in mm^2^ per second. Sway length (sway path) means the length of the trajectory followed by the CoP normalized to the duration of acquisition; is coincides with the mean velocity or average speed [31].

Our study analysing 95 healthy young adults showed that the confidence ellipse area was lower in maximum intercuspation in comparison to mandibular postural position. Perinetti recommended future investigations that should focus on the effects of extreme mandibular positions [9]. In our study we also recorded the stabilometric parameters in maximum mouth opening. The stabilometric parameters, CoP path length and maximum CoP speed, were significantly greater in maximum mouth opening condition in comparison to mandibular postural position, maximum intercuspation and biting on cotton rolls position. The confidence ellipse area was also higher in maximum mouth opening when compared to maximum intercuspation. These data suggest that the postural stability, expressed as CoP path length, maximum CoP speed and confidence ellipse area, is most decreased when the examination was performed in maximum mouth opening condition.

Baldini et al. (2013) assessed 44 healthy volunteers in different conditions (mandibular postural position, mandibular position of centric occlusion and mandibular position with 8-mm thick cotton rolls) and noticed that the position of the centre of the foot pressure was not influenced by occlusal components [5]. The current study showed that there were no differences in the CoP displacement measurement when comparing the mandibular postural position, maximum intercuspation, biting on cotton rolls and maximum mouth opening conditions.

The study of Oie E. et al. examining 15 adult males showed that occlusal contact is one of the factors that affect gravity fluctuation; appropriate occlusion attained by maintaining even occlusal contact in the posterior region is crucial for gravity fluctuation [32]. Julia-Sanchez S. et al. (2016) showed that mandibular position has a significant influence in the balance control; the body balance was better when dental occlusion was set in cotton rolls mandibular condition (with 8 mm thick cotton rolls placed between the two dental arches) in comparison with intercuspal position (achieved by clenching the teeth) [33]. Scharnweber et al. (2016) after assessing 87 healthy male subjects noticed that blocking occlusion leads to sway reduction [34]. Our study recorded that in maximum intercuspation, the stabilometric parameters showed a better postural stability than in mandibular postural position.

The lack of comparison between the subjects without any composite fillings and the subjects with one or more composite fillings is a limitation of our study.

## Conclusions

In right-handed young adults the static plantar pressure is influenced by the maximum mouth opening. This dental condition was characterized by a reduced loading on the left calcaneus in comparison to maximum intercuspation and an increased loading on the left 5^th^ metatarsal head in comparison to maximum intercuspation and biting on cotton rolls. Regarding the stabilometry, an improved postural stability was recorded in maximum intercuspation in comparison to mandibular postural position. The maximum intercuspation is used by the subject during swallowing, while the mandibular postural position allows the relaxation of the masticatory muscles after functional moments. In contrast, a decreased postural stability was recorded when the examination was performed with maximum mouth opening.

## Supporting information

S1 Fig (TIF)
