# Supplementary figures and images for: Do different dental conditions influence the static plantar pressure and stabilometry in young adults?
Source: PLoS One. 2020 Feb 11;15(2):e0228816. doi: 10.1371/journal.pone.0228816 (PMC7012393; doi:10.1371/journal.pone.0228816)

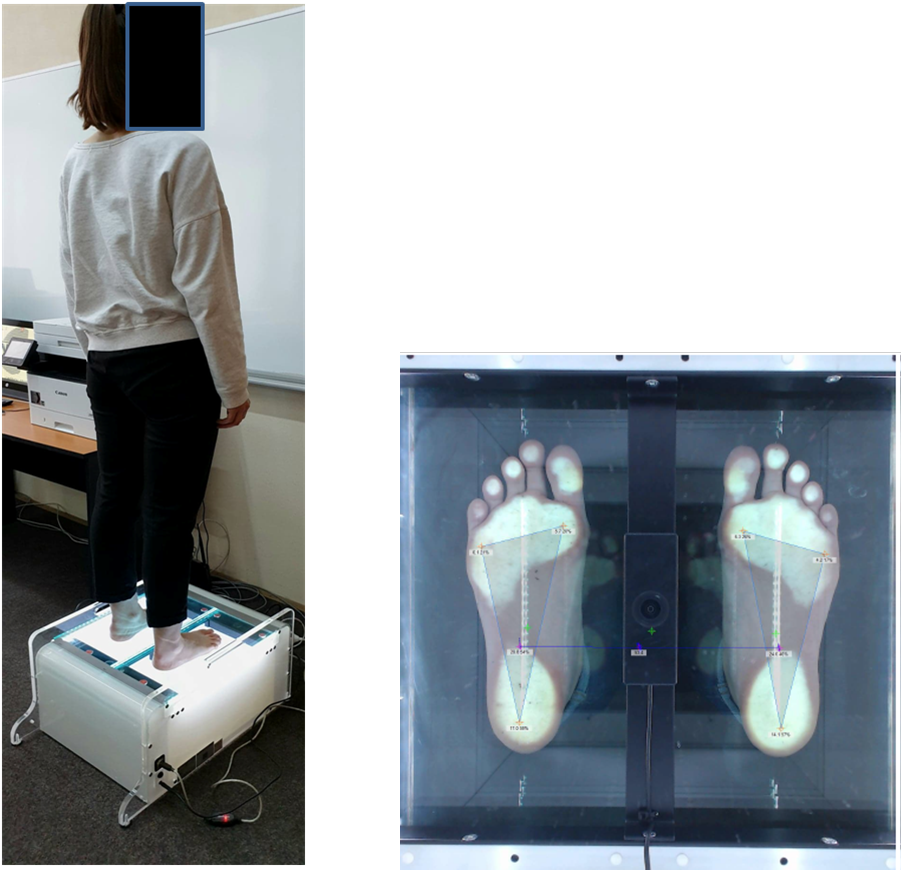

Supplement: S1 Fig — (TIF) [file pone.0228816.s001.tif]
